# Supplementary material for: Prediction of Intraparenchymal Hemorrhage Progression and Neurologic Outcome in Traumatic Brain Injury Patients Using Radiomics Score and Clinical Parameters
Source: Diagnostics (Basel). 2022 Jul 10;12(7):1677. doi: 10.3390/diagnostics12071677 (PMC9320220; doi:10.3390/diagnostics12071677)
Supplement: Supplementary file 1 [file diagnostics-12-01677-s001.zip › diagnostics-1763415-supplementary.pdf]

Supplemenary

Table S1. ICC for selected radiomic features based on manual segmentation results of the same reader twice and two independent readers.

	ICC	
**Selected Feature**	**Reader A twice**	**Reader A and B**
original_glcm_Autocorrelation	0.8637	0.6405
original_glcm_ClusterShade	0.9768	0.9100
original_glcm_Imc2	0.9589	0.9087
original_glcm_InverseVariance	0.9904	0.9781
original_glrlm_RunEntropy	0.9833	0.9561
original_glrlm_RunLengthNonUniformityNormalized	0.9710	0.9325
original_glszm_GrayLevelNonUniformityNormalized	0.7879	0.4226
original_glszm_SmallAreaHighGrayLevelEmphasis	0.9127	0.7997
original_glszm_ZoneEntropy	0.9526	0.8470

ICC, intraclass correlation coefficient.
